# The efficacy and safety of CD7 chimeric antigen receptor T-cell therapy for hematologic malignancies: a systematic review and meta-analysis

**DOI:** 10.3389/fonc.2024.1478888

**Published:** 2025-01-07

**Authors:** Jile Liu, Yuxin An, Rui Sun, Xiaomei Zhang, Shujing Guo, Xuejin Gao, Mingfeng Zhao

**Affiliations:** ^1^ First Center Clinical College, Tianjin Medical University, Tianjin, China; ^2^ Nankai University School of Medicine, Nankai University, Tianjin, China; ^3^ Department of Emergency, Tianjin First Central Hospital, Tianjin, China; ^4^ Department of Hematology, Tianjin First Central Hospital, Tianjin, China

**Keywords:** CD7 CAR-T cell, chimeric antigen receptor T cells, T-lymphocyte hematologic malignancies, gene editing, allogeneic stem cell transplantation

## Abstract

**Introduction:**

CD7 chimeric antigen receptor T-cell (CAR-T cell) therapy is an emerging method for treating hematological malignancies, and is another breakthrough in CAR-T cell therapy.

**Methods:**

This study summarizes the currently published clinical research results on CD7 CAR-T cells and evaluates the safety and effectiveness of CD7 CAR-T cell therapy.

**Results:**

Among the 13 studies included in this study, a total of 200 patients received CD7 CAR-T cell therapy, including 88 patients who received autologous CAR-T cells, 112 patients who received donor derived CAR-T cells. 87% (80% -94%, *I^2^* =29.65%) of patients achieved complete remission. The incidence of cytokine release syndrome (CRS) was 94% (88% -98%, *I^2^* =32.71%, p=0.12), while the incidence of severe CRS (grade ≥ 3) was 12% (5% -20%, *I^2^* =41.04%, p=0.06). As for the incidence of immune effector cell-associated neurotoxicity syndrome (ICANS), it is 4% (1% -7%, *I^2^* =0, p=0.72). Through analysis of the key clinical issues, we found that consolidation allogeneic hematopoietic stem cell transplantation (allo-HSCT) after CAR-T cell therapy can significantly improve survival and avoid recurrence. Therefore, we believe that the consolidation allo-HSCT after CD7 CAR-T cell therapy should be advocated. And patients who received CD7 CAR-T cell therapy without gene editing had significantly longer overall survival than those who received CD7 CAR-T cell therapy with gene editing. This suggests that gene edited CD7 CAR-T cells may pose some potential risks that limit the long-term survival of patients.

**Conclusion:**

Our study confirms the efficacy and safety of CD7 CAR-T cells and provides research directions for the subsequent treatment.

**Systematic Review Registration:**

https://www.crd.york.ac.uk/PROSPERO/display_record.php?RecordID=502896, identifier CRD42024502896.

## Introduction

Acute T-lymphoblastic leukemia accounts for approximately 15% of childhood acute lymphoblastic leukemia (ALL) and 25% of adult ALL (1). T-cell lymphoblastic lymphoma (T-LBL) is diagnosed when T lymphoblastic cells present as a mass but do not infiltrate the bone marrow. The two diseases are similar, both caused by lymphoblasts. The disease molecular markers in T-LBL and T-ALL patients are basically consistent, which both have high invasiveness. The overall survival (OS) rate of pediatric T-ALL patients at 5 years is about 63% and that of adult patients is 40% to 55% (2) (3). Moreover, T-ALL recurrence is mostly early (within 2 years after diagnosis) (4). After recurrence, the prognosis of T-ALL patients is extremely poor. The OS rate in children with recurrent T-ALL is less than 25% (5). The 5-year OS rate of adult T-ALL patients is only 7% (6). The proportion of recurrent T-ALL patients achieving complete remission (CR) again is significantly reduced. At present, the treatment options for T-ALL/LBL (especially relapsing/refractory T-ALL/LBL) are relatively limited, mainly relying on high intensity chemotherapy and hematopoietic stem cell transplantation, which need to be optimized (7).

With the rise of immunotherapy in the field of tumor treatment, CAR-T cell therapy has received great attention from researchers and healthcare workers. The preparation of CAR-T cells usually requires collecting peripheral blood mononuclear cells (PBMCs) from patients or donors, selecting T cells from them, and activating T cells with CD3/CD28 magnetic beads (8). And methods such as lentivirus or electroporation are used to make the surface of T cells carry CAR structures that can recognize tumor specific targets, which makes it different from T cell receptor-T (TCR-T) cells and breaks free from major histocompatibility complex (MHC) limitations, thus allowing for broader application space (9). The single-chain variable fragment (scfv) confers CAR-T cells with precise identification capability, whereas the intracellular signaling domains can trigger CAR-T cell-induced cytotoxicity (10). In the field of B-cell malignancies, CD19 CAR-T cell therapy has achieved remarkable success and sparked the enthusiasm of researchers to explore more targets for the treatment of other hematological malignancies and solid tumors. The potential for CAR-T cell therapy to revolutionize cancer treatment is immense.

Up till now, three targets have received attention among numerous targets in hematological tumors and relevant clinical trials have been widely conducted in multiple centers. They are CD19 CAR-T cells used for the treatment of B-cell malignancies, BCMA CAR-T cells used for the treatment of multiple myeloma, and CD7 CAR-T cells used for the treatment of T-lymphocyte hematologic malignancies and some acute myeloid leukemia (AML). Following extensive research proving their efficacy and safety, the Food and Drug Administration (FDA) has now authorized CAR-T cell products that target the first two targets (11–13). Therefore, using a systematic review and meta-analysis of previous published cases, our study investigates the safety and efficacy of CD7 CAR-T cells. We included 13 clinical studies with 200 patients who received CD7 CAR-T cell products (**Table 1**). Among the 13 studies, a total of 10 reported relatively accurate survival times, involving 112 patients. Therefore, we conducted subgroup analyses based on 112 patients who reported survival time and evaluated the differences in OS and progression free survival (PFS) using Kaplan Meier survival curves.

**Table 1:** Clinical trial related data of 13 CD7 CAR-T cell products.

Name	Trial	Patients (n=)	Diagnosis	Conditioning	Source	Dosage	Clinical efficacy	Adverse reactions	Early causes of death (the first 100 days)
Mingzhi Zhang (32, 33)	NCT04004637	8	r/r T-ALL/LBL n=5 r/r ETP-ALL/LBL n=3	FC	Autologous	1.0x10 ^6^/kg (n=5) 1.5x10^6^/kg (n = 1) 2x10^6^/kg (n = 2)	CR (7/8) PR (1/8)	7/8 grade 1-2 CRS, 1/8 grade 4 CRS; 2/8 grade 4 thrombocytopenia, 7/8 grade 3-4 neutropenia 2/8 grade 2 anemia	1 CR MRD-patient died in the abdominal infection within the first 100 days
Yongxian Hu (19)	NCT04538599	12	r/r T-ALL/LBL n=11 r/r AML n=1	FCE	Universal donor	(1-3) x10^7^/kg	ORR (9/11) CR (7/11)	10/11 grade 1-2 CRS	1 patient died of sepsis 24 days after receiving CD7 CAR-T cell infusion 1 CR patient died from relapse within the first 100 days. A non-relieved patient died within the first 100 days.
Xian Zhang (34)	NCT04620655	10	r/r T-ALL n=7 r/r T-LBL n=3	FCE	Universal donor	(0.5-1) x10^7^ /kg (n=3) 2x10^7^ /kg (n=6) 4x10^7^ /kg (n=1)	CR (7/10 MRD-CR, 1/10 MRD+CR)	9/10 grade 1 CRS, 1/10 grade3 CRS, n=1 grade3 ICANS	1 CR patient died from bacterial infection on day 35
Yue Tan (35)	NCT04689659	20	r/r T-ALL n=20	FC	Donor	(0.5-1) x10^6^/kg	ORR (18/20)	2/20 grade 3-4 CRS, 8/20 grade 1-2 GVHD, 3/20 SAE	/
Shiqi Li (36)	ChiCTR1900025311	12	r/r T-ALL n=11 r/r T-LBL n=1	FC+mefalam/etoposide/prednisone	Universal donor	(6.2-8.6) x10^6^ /kg (n=3) (1-1.5) x10^7^ /kg (n=9)	CR or CRi (11/12)	2/12 grade 2 CRS, 8/12 grade 3 CRS, none ICNAS	/
Xian Zhang (37)	NCT04572308 NCT04916860	60	r/r T-ALL n=35 r/r T-LBL n=25	FC	Autologous (n = 58) Donor (n = 2)	5x10^5^ /kg (n=25) (1-1.5) x10^6^ /kg (n=34) 2x10^6^ /kg (n=1)	CR (51/54 MRD-CR, 1/54 MRD+CR) NR (2/54)	42/60 grade 1 CRS, 6/60 grade 2 CRS, 6/60 grade 3 CRS; 2/60 grade 1 ICNAS, 1/60 grade 4 ICANS 10/60 grade 3 infection	/
Yue Tan (38)	ChiCTR2000034762	20	r/r T-ALL n=20	FC	Donor	1x10^6^ (± 30%) /kg	ORR (19/20) CR (17/20)	18/20 grade 1-2 CRS, 2/20 grade 3-4 CRS; 3/20 grade 1-2 ICANS; 20/20 ≥grade 3 cytopenia	1 patient had CD7 negative recurrence. 1 patient died after stem cell transplantation.
Wei Chen (39)	ChiCTR2200058969	7	r/r T-LBL n=4 r/r T-ALL n=3	FC	Donor	1x10^5^/kg	CR (7/7)	5/7 grade1-2 CRS, 1/7 grade 3 CRS; 1/7 grade 4ICANS; 2/7 GVHD	No early death patients.
Yinqiang Zhang (40)	NCT04823091	10	r/r T-ALL n=5 r/r T-LBL n=5	FC	Donor (Donors must be equal to or over 5/10 HLA-identical siblings or 10/10 HLA-matched unrelated donors) n=5 Autologous n=5	1x10 ^6^/kg (n=5) 2x10^6^/kg (n = 5)	ORR (7/10, 3/7 autologous, 4/7 donor); CR (7/7 BM MRD- CR)	7/10 grade 1-2 CRS, 1/10 grade 3 CRS; 2/10 grade1-2GVHD; 10/10 grade 4 cytopenia	No early death patients.
Xian Zhang (41)	NCT04938115	11	r/r MPAL n=11	FC	Donor n=1 Autologous n=10	(1-5) x10 ^5^/kg (n=4) 1.0x10^6^/kg (n = 7)	CR (10/11 MRD-CR)	8/11 grade1 CRS, 2/11 grade 2 CRS, 1/11 grade3 CRS; none ICANS	/
Jiali Cheng (42)	NCT06136364	7	r/r T-LBL n=5 r/r T-ALL n=2	FC	Autologous	1x10 ^6^/kg (n=4) 2x10^6^/kg (n = 3)	CR (7/7)	5/7 grade1-2 CRS 1/7 grde3 CRS; 7/7 grade4 PHT	No early death patients.
Armin Ghobadi (43)	NCT04984356	18	r/r T-ALL n = 11 r/r T-LBL n = 7	FC	Universal donor	DL1: 3 /18 1×10^8^, DL2: 3/18 3×10^8^, DL3: 6/18 6×10^8^, DL4: 6/18 9×10^8^	DL1: NR; DL2-3: 7/12 MRD- CR	13/18 grade 1-2 CRS, 1/18 grade 3 CRS; 1/18 grade1 ICANS; none GVHD, PHT	/
Fang Liu (44)	NCT05454241	5	r/r T-LBL n=1 r/r T-ALL n=4	FC	Universal donor	2x10 ^6^/kg	CR (4/5 MRD-CR), PR (1/5)	4/5 grade1-2 CRS	No early death patients.

ALL, acute lymphoblastic leukemia; LBL, lymphoblastic lymphoma; MPAL, mixed phenotype acute leukemia; AML, acute myelocytic leukemia; r/r, relapse/refractory; CR, complete response; CRi, complete remission with incomplete recovery; PR, partial response; NR, non-remission; ORR, objective response rate; MRD, minimal residual disease; CRS, cytokine release syndrome; ICANS, immune effector cell-associated neurotoxicity syndrome; SAE, serious adverse event; GVHD, graft versus host disease; PHT, prolonged hematologic toxicity; BM, bone marrow; FC, fludarabine and cyclophosphamide; FCE, fludarabine, cyclophosphamide and etoposide.

During the application of CD7 CAR-T cell therapy, some issues have arisen. Researchers have proposed some potential treatment options for these issues. Firstly, the fratricide phenomenon in CD7 CAR-T cell therapy. Research has shown that more than 95% of T-lymphocyte hematologic malignancies (including T-ALL and T-LBL) express CD7 on the surface, making it a good therapeutic target (14). Meanwhile, approximately 30% of patients with acute myeloid leukemia have high expression of CD7 in their tumor cells (15). However, due to the expression of CD7 on the surface of CAR-T cells, fratricide often occurs, which affects the efficacy of CD7 CAR-T cells. In order to avoid the occurrence of self-killing behavior, researchers have proposed two types of strategies. One method is to use gene-editing techniques (including base editors) to knock out the CD7 gene of CD7 CAR-T cells so that the CD7 protein is not expressed on the cell membrane (16, 17). Another method is to reduce fratricide of CD7 CAR-T cells without gene editing techniques, such as utilizing a CD7 protein expression blocker (PEBL) and naturally selecting T cell (18). In addition, some other methods for preparing CD7 CAR-T cells have also been used in preclinical and clinical trials. Secondly, because of the unique biology of T-lymphocyte hematologic malignancies, it is hard to get a lot of healthy cells from patients. This makes choosing autologous or donor T cells even more important. At the moment, a lot of studies are using gene editing to remove the TRAC and CD52 genes from healthy donor T cells. This is done to create universal CD7 CAR-T cells that don’t have graft-versus-host disease (GVHD), which achieve good therapeutic effects (19). In addition, how to avoid recurrence in patients receiving CD7 CAR-T cell therapy is an important issue. Consolidating allo-HSCT after CD19 CAR-T cell therapy is a good strategy for prolonging patient survival and avoiding recurrence (20). We are curious whether consolidating allo-HSCT after CD7 CAR-T therapy can achieve the same good therapeutic effect as consolidating transplantation after CD19 CAR-T cell therapy.

**Figure 1:**
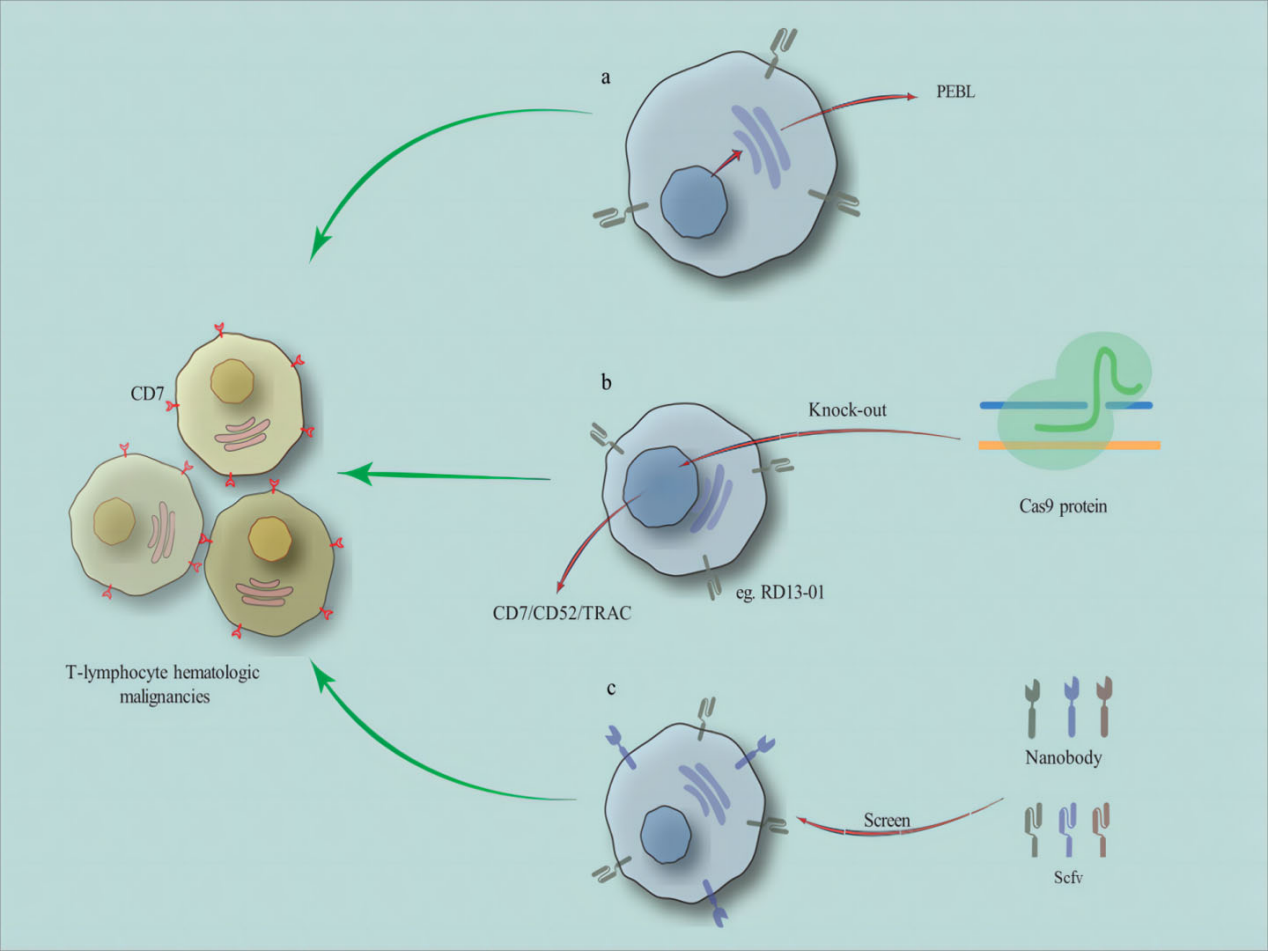
The related methods for preparing CD7 CAR-T cells. **(A)** reducing fratricide of CD7 CAR-T cells without gene editing techniques, such as utilizing a CD7 protein expression blocker (PEBL). (as well as natural selection of T cells and other methods) **(B)** using gene editing (eg. CRISPR-Cas9) to remove the TRAC and CD52 genes from healthy donor T cells to prepare universal CD7 CAR-T cells. **(C)** the CAR structures include nanobody-derived or scFv-derived.

Emerging technologies such as nanobody-derived CAR-T cells are also challenging the position of traditional scFv-derived CAR-T cells. Unlike scFv with two variable domains of variable heavy chain (VH) and variable light chain (VL), nanoantibodies derived from alpacas and sharks only have the VHH domain, which is not only small in structure but also very stable (21). Compared to traditional scFv derived CAR-T cells, CAR-T cells prepared by emerging technologies such as nanoantibodies may have more potential (**Figure 1**).

Therefore, we also focused on exploring the clinical hotspots of CD7 CAR-T cells. Through subgroup analysis, we evaluated effectiveness of the therapy using different survival curves, including consolidating transplantation or not, gene-editing or non-gene-editing CAR-T cells, autologous or allogeneic CAR-T cells, nanobody-derived or scFv-derived CAR-T cells, providing some assistance for subsequent CD7 CAR-T therapy research.

## Methods

### Searching strategy and selection criteria

**Figure 2:**
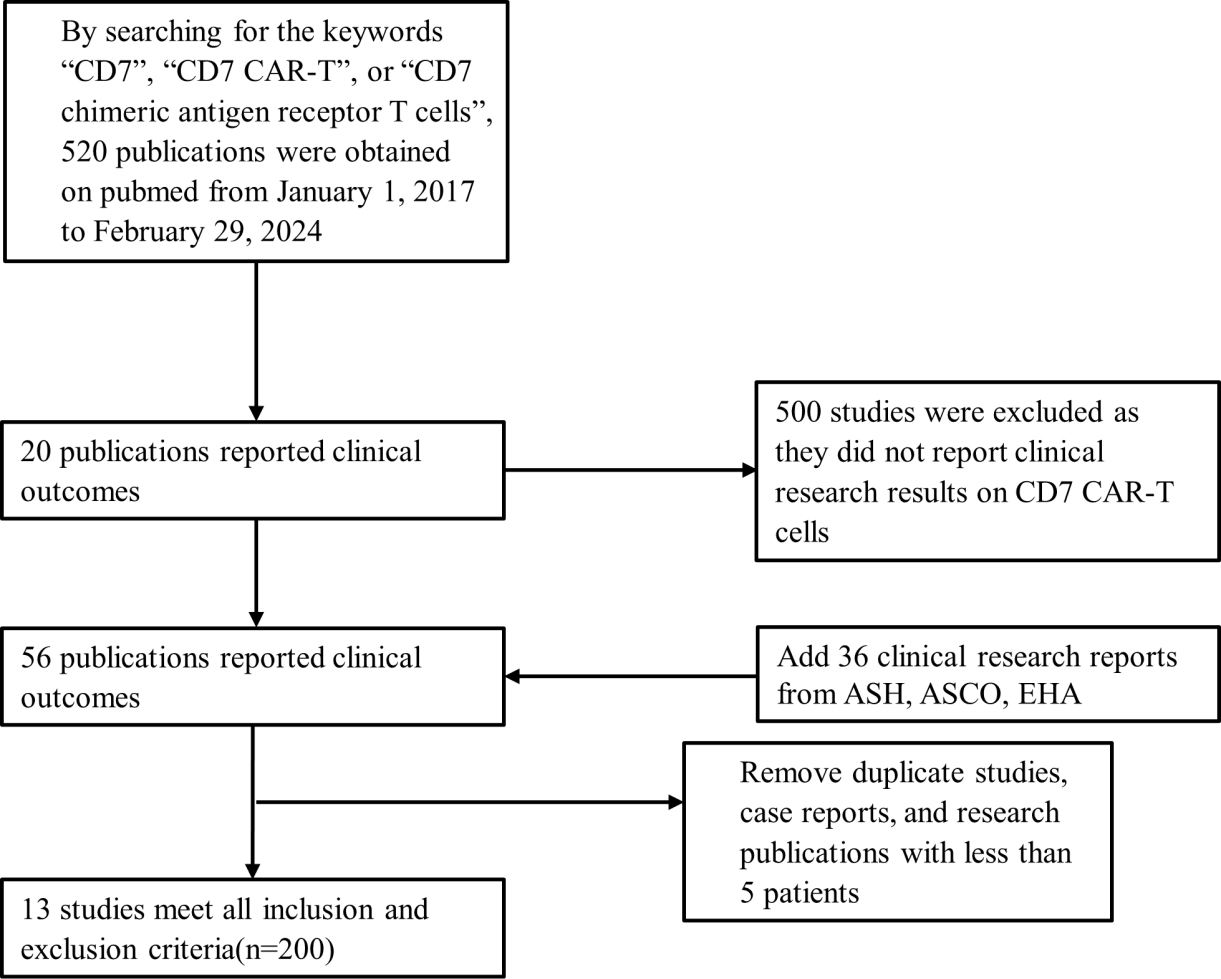
Searching strategy and study selection. CAR-T, chimeric antigen receptor of T cell; ASH, American Society of Hematology; ASCO, American Society of Clinical Oncology; EHA, European Hematology Association.

Our study is the first systematic review and meta-analysis of the safety and efficacy of CD7 CAR-T cell therapy for hematological malignancies in the world. The relevant clinical studies included in this study were retrieved through PubMed and conference abstracts published by the American Society of Clinical Oncology (ASCO), American Society of Hematology (ASH), and European Hematology Association (EHA). All clinical studies were registered on Clinicaltrials.gov (NCTnumber) or the Chinese Clinical Trial Registry (ChiCTRnumber). The searching keywords are “CD7”, “CD7 CAR-T”, or “CD7 chimeric antigen receptor T cells”. Due to the first public report on CD7 CAR-T cell therapy occurring in 2017, the searching time of this study was set from January 1, 2017 to February 29, 2024. Patient data was completely extracted from publicly available clinical reports, there were no additional requirements for raw patient data for the authors of these studies. Database searching and data collection were independently conducted by two authors (Jile Liu and Yuxin An). If there is no consensus on opinions, another author (Rui Sun) will determine whether to omit or retain it. The PRISMA fow diagram in **Figure 2** describes the searching strategy followed by this study.

### Data analysis

We have retrieved a total of 13 clinical research reports on the treatment of hematological malignancies with CD7 CAR-T products. Based on the clinical trial registration number, main author and affiliation, as well as CD7 CAR-T product types, independent clinical studies were identified. Also, clinical studies and case reports with duplicate data and small sample sizes were excluded. For example, clinical studies on base-edited CD7 CAR-T cells with only three cases were excluded.

We evaluated the CR, CRS, and ICANS of various studies. Due to the diversity between the studies, we adopted a random effects model. Heterogeneity was determined by the forest plots and *I^2^*. The results are reported with a 95% confidence interval (CI). Subgroup analyses were conducted to assess the differences between each group of studies. P values were calculated based on the heterogeneity between subgroups. By subgrouping whether to consolidate transplantation after infusion, gene-editing or non-gene-editing, donor or autologous derived T cells, as well as scFv-derived or nanobody-derived CAR structure, the differences in OS and PFS were evaluated using Kaplan-Meier survival curves. The forest plots are made by Stata 14.0, and the Kaplan-Meier survival curves are plotted by GraphPad Prism 8.0. This study has been registered with PROSPERO (CRD42024502896).

## Results

### Analysis of the efficacy and safety of CD7 CAR-T cells

**Figure 3:**
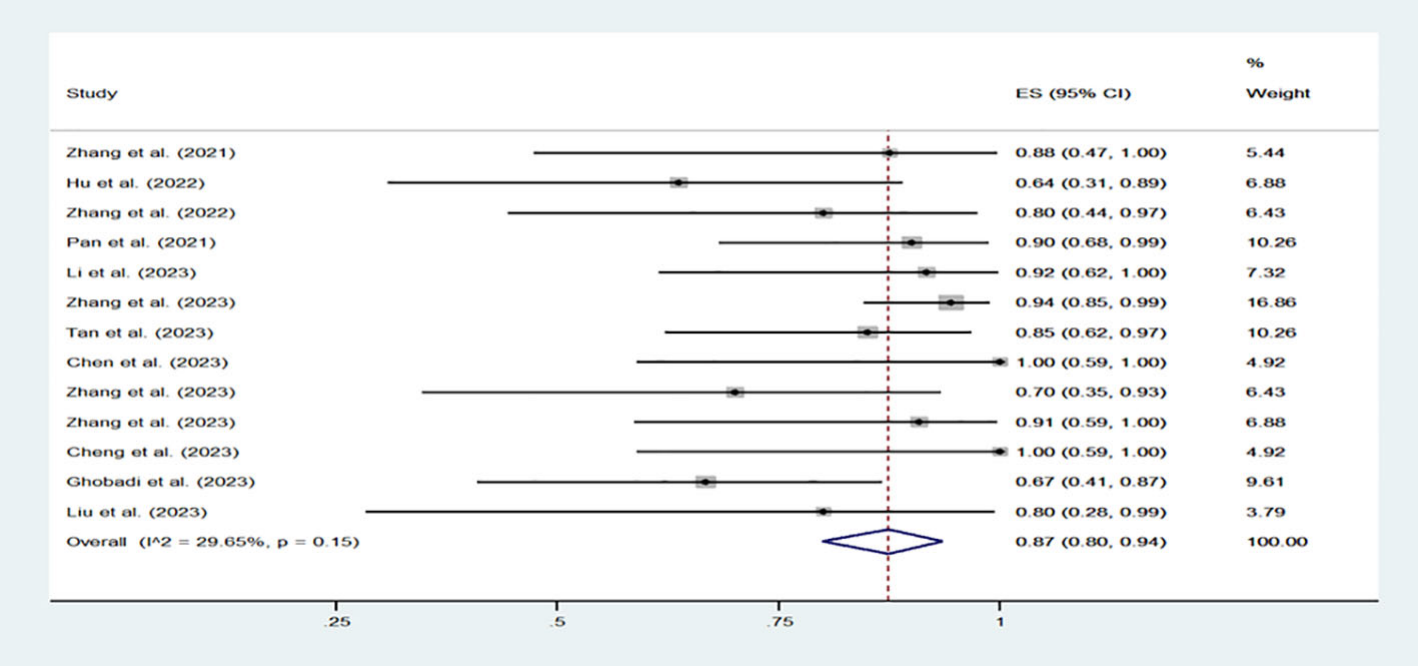
Forest plot of clinical efficacy for patients in 13 CD7 CAR-T clinical trials.

A total of 13 clinical trial reports were included, involving 200 patients receiving CD7 CAR-T cell therapy. Among the 200 patients, except for 1 patient with AML and 11 patients with mixed phenotype acute leukemia (MPAL), all other patients had T lymphocyte hematological malignancy. All patients were diagnosed with recurrent/refractory hematological malignancies and received multi line treatment. All patients meet the CD7 CAR-T trial criteria. **Table 1** provides a detailed presentation of the patient baseline, clinical efficacy, and side effects reported in various clinical reports. 167 patients achieved CR, with a rate of 87% (80%-94%, I^2^ = 29.65%, p=0.15). (**Figure 3**) The CR rate of T lymphocyte hematological malignancies was 83% (156/188), while the CR rate of AML and MPAL were 92% (11/12). Regarding the safety of CD7 CAR-T cells, we evaluated the common side effects of CRS and ICANS after CD7 CAR-T cell infusion. CRS, the most common adverse effect of CAR-T treatment, presents with symptoms such as fever, fatigue and anorexia. It can be assessed by detecting the level of various cytokines in the patient’s serum, including interleukin-6/10, interferon-γ and so on (22). Severe CRS (grade ≥ 3) often affect the survival and prognosis of patients. ICANS, the second most likely side effect after CAR-T cell therapy, is characterized by aphasia, changes in consciousness levels, impaired cognitive function, epilepsy, and cerebral edema (23). Among the 200 patients included in the study, the incidence of CRS was 94% (88% -98%, I^2^ = 32.71%, p=0.12), while the incidence of severe CRS (grade ≥ 3) was 12% (5% -20%, I^2^ = 41.04%, p=0.06). (**Supplementary Figures 1A, B**) As for the incidence of ICANS, it is 4% (1% -7%, I^2^ = 0, p=0.72). (**Supplementary Figure 1C**) Therefore, combining the above statistical data, we can conclude that CD7 CAR-T cell therapy for hematologic malignancies is a safe and effective treatment approach. Perhaps in the near future, we can choose to use CD7 CAR-T cell therapy earlier in the treatment of T lymphocyte malignancies and AML patients with high CD7 expression.

### Subgroup analysis

Based on four major clinical practice controversies—including whether consolidation allo-HSCT was used after CD7 CAR-T cell therapy, whether gene-editing methods were used to make CD7 CAR-T cells, the source of T cells used to make CAR-T cells, and whether CAR-T cells were nanobody-derived or single chain variable regions (scFv)-derived—we compared OS and PFS between groups based on cases that had been published.

**Figure 4:**
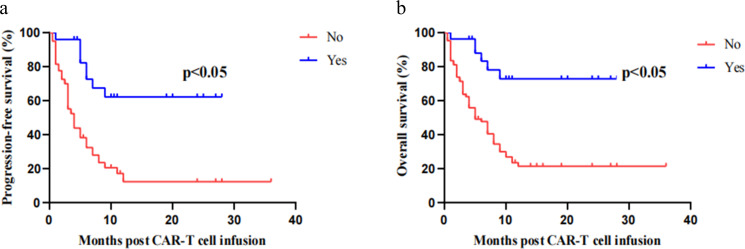
Kaplan–Meier progression-free survival curves and overall survival curves. Patients who consolidated allo-HSCT after CD7 CAR-T cell therapy showed significant improvements in OS **(A)** and PFS **(B)**.

An increasing number of studies indicate that consolidating allo-HSCT after receiving CAR-T cell therapy is a significant therapeutic approach to prevent recurrence and prolong disease-free survival in patients (24). Our study included 27 cases of consolidation allogeneic hematopoietic stem cell transplantation after CD7 CAR-T cell therapy (25). Through subgroup analysis with non-transplant patients, it was found that those who consolidated allo-HSCT showed significant improvements in OS and PFS, suggesting that consolidating allo-HSCT after CD7 CAR-T cell therapy is an active treatment option for prolonging patient survival and consolidating therapeutic efficacy (**Figures 4A, B**).

The application of gene-editing to avoid fratricide and GVHD is currently one of the mainstream methods for preparing CD7 CAR-T cells (17). However, genetic toxicity is an issue that cannot be ignored in gene-editing. Although there are currently no reports proving the potential threat of gene edited CAR-T cells, we still need to evaluate the safety of gene edited CD7 CAR-T cells. Therefore, we evaluated the survival of gene-edited CD7 CAR-T cells. Among the included cases, survival curve analysis was performed on the OS and PFS of 60 patients who received non-gene-edited CD7 CAR-T cell therapy and 52 patients who received gene-edited CD7 CAR-T cell therapy to determine whether there were statistical differences. The Kaplan-Meier survival curve showed that there was no statistically significant difference in PFS between patients receiving non-gene-edited CD7 CAR-T cells and gene-edited CD7 CAR-T cells. However, the OS of patients receiving non-gene-edited CD7 CAR-T cell therapy was longer than the other group, and the difference was statistically significant (P=0.016). Therefore, we believe that there may be potential threats that may occur in patients receiving gene edited CD7 CAR-T cell therapy (**Supplementary Figures 2A, B**).

Due to the patient’s malignant tumor of T lymphocytes, there is a risk of tumor contamination of patients’ peripheral blood T cells. Meanwhile, after multiple rounds of chemotherapy, the quality of T cells in patients generally deviates from that of healthy individuals. Therefore, in the field of CD7 CAR-T cells, it is more common to use donor and healthy human-derived T cells to prepare CD7 CAR-T cells than CD19 CAR-T cells and BCMA CAR-T cells. Therefore, based on existing data, we divided into subgroups for Kaplan Meier survival. We found that there was no statistically significant difference both OS and PFS in the existing data (**Supplementary Figures 2C, D**).

Research has found that compared to traditional scFv-derived CARs, nanobody-derived CARs have greater advantages (23). First, nanobody-derived CAR-T cells are not easily exhausted. Second, nanobody-derived CAR-T cells have low immunogenicity, avoiding the production of related antibodies in the body. Third, the smaller structure of CAR makes it easy to produce CAR-T and is beneficial for the preparation of dual target CAR-T cells (23). Among the 13 studies we included, only 2 clinical studies reported using nanobody-derived CD7 CAR-T cells to treat patients. A total of 15 patients received nanobody-derived CD7 CAR-T therapy, of which 14 patients achieved CR. We conducted a subgroup survival analysis and discovered no statistically significant differences in OS and PFS between patients who received scFv-derived and nanobody-derived CD7 CAR-T cells. (**Supplementary Figures 2E, F**).

## Discussion

Our study confirms the clinical efficacy and safety of CD7 CAR-T cells in patients with hematologic malignancies through systematic review and meta-analysis. Among the 200 patients included in this study, the CR rate after CD7 CAR-T cell infusion was as high as 87%, with only an 8% and 4% probability of severe CRS and ICANS affecting prognosis. Due to the small number of patients receiving CD7 CAR-T cell therapy and a lack of specific data from various conference sources, we combined patients with T-cell malignancies and AML patients to conduct a comprehensive evaluation of the effectiveness and safety of CD7 CAR-T cells. We suggest that in the future, when more comprehensive data is available, separate evaluations should be conducted for patients with T-cell malignancies and AML patients.

At the same time, we also analyzed the issues that clinical doctors are more concerned. Through the analysis of the survival curve, consistent with the conclusion of Li Z et al. (25), consolidation allo-HSCT after CD7 CAR-T cell therapy can significantly improve OS and PFS. This conclusion is of great significance for clinical guidance. We suggest that for patients assessed as suitable for allo-HSCT after CD7 CAR-T cell infusion, allo-HSCT should be recommended to consolidate and improve treatment outcomes. This is also beneficial for the recovery of T cells in patients after CD7 CAR-T therapy.

In addition, we found that patients treated with CD7 CAR-T cells prepared using gene-editing technology had significantly lower overall survival compared to patients treated with CD7 CAR-T cells without gene-editing, although there was no significant difference in progression free survival between the two groups. We believe that this may be due to the potential genotoxic effects of gene-editing technology. For example, one of the common toxic side effect of gene-editing is the “off-target” effect. Due to the possibility of random similarity between base sequences at other positions of DNA and the target gene, if the cleavage enzyme identifies sites incorrectly, new genes may be knocked into other positions, leading to gene editing errors. It is worthy our attention. We should actively explore measures to reduce the risk and harm of genetic toxicity. The emergence of base editing technology is also worth looking forward to see whether it can reduce the risk of gene toxicity in the field of universal CAR-T cells (26). For the selection of autologous and donor T cells, we found no statistically significant difference in OS and PFS between the two groups, which is consistent with the conclusion of Zhang YQ et al. Therefore, when dealing with patients who have undergone multiple rounds of chemotherapy and have poor T cell status, we can choose donor T cells to prepare CD7 CAR-T cells to improve the success rate of preparation. For the nanobody-derived CD7 CAR-T cells, survival analysis did not show any statistical differences compared to traditional CAR-T cells. Although some preliminary conclusions have been drawn from this study, the number of patient cases is still relatively small. We look forward to more research reports being published in the future to obtain more accurate results and guide clinical applications.

At present, the CAR-T immunotherapy treating T-lymphocyte hematologic malignancies field is developing rapidly. Studies have reported the activity of dual target CAR-T against T-lymphocyte hematologic malignancies, such as CD5/CD7 CAR-T cells (27). Meanwhile, in the field of basic research, there are other options for preparing CD7 CAR-T cells, such as selecting CD7 negative T cells to prepare CAR-T cells and using antibodies to reduce the occurrence of fratricide effects during *in vitro* preparation (28, 29). Studies have shown that approximately 30% of patients with AML have CD7 antigen expression on leukemia cells (15, 30). Lu Y et al. have validated the inhibitory effect of CD7 CAR-T cells on CD7 positive AML tumors *in vitro* experiments and animal models (15). Currently, there are many studies on targets of CAR-T cell therapy for patients with refractory/relapsed AML, such as CLL1, CD123 etc. (31) However, these CAR-T cells often lead to severe neutropenia, which can easily lead to infection and affect patient prognosis (15). CD7 is not expressed in normal myeloid cells, so CD7 CAR-T cells will not lead to severe neutropenia. Therefore, we suggest that for AML patients with strong positive CD7 antigen expression, treatment with CD7 CAR-T cells can be attempted. We can believe that CD7 CAR-T products play a significant role in the treatment of hematologic malignancies with high malignancy and limited treatment options.

## Data Availability

The original contributions presented in the study are included in the article/**Supplementary Material**. Further inquiries can be directed to the corresponding authors.
