# Supplementary figures and images for: The efficacy and safety of CD7 chimeric antigen receptor T-cell therapy for hematologic malignancies: a systematic review and meta-analysis
Source: Front Oncol. 2025 Jan 7;14:1478888. doi: 10.3389/fonc.2024.1478888 (PMC11752923; doi:10.3389/fonc.2024.1478888)

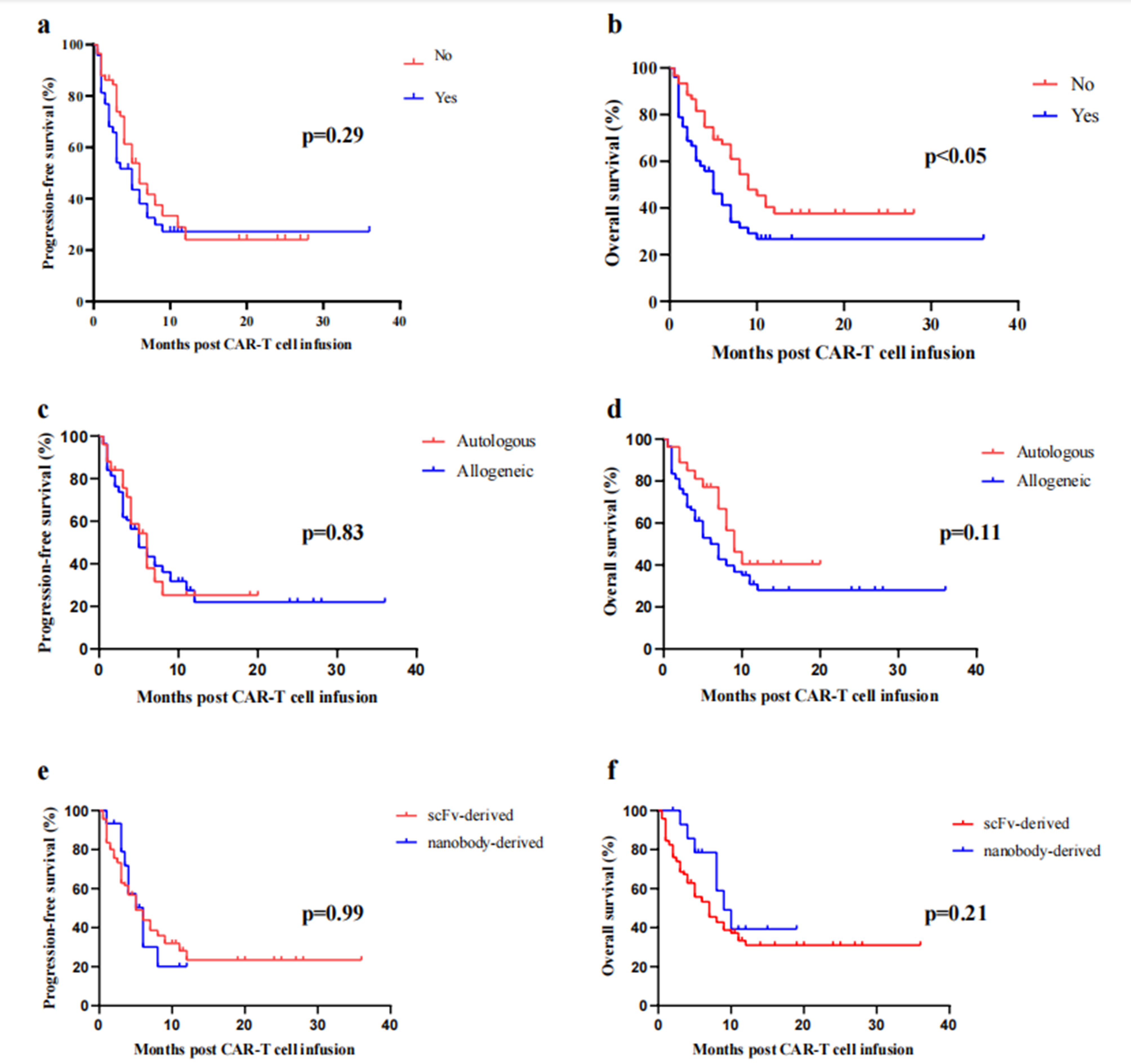

Supplement: Supplementary file 1 [file Image1.tif]

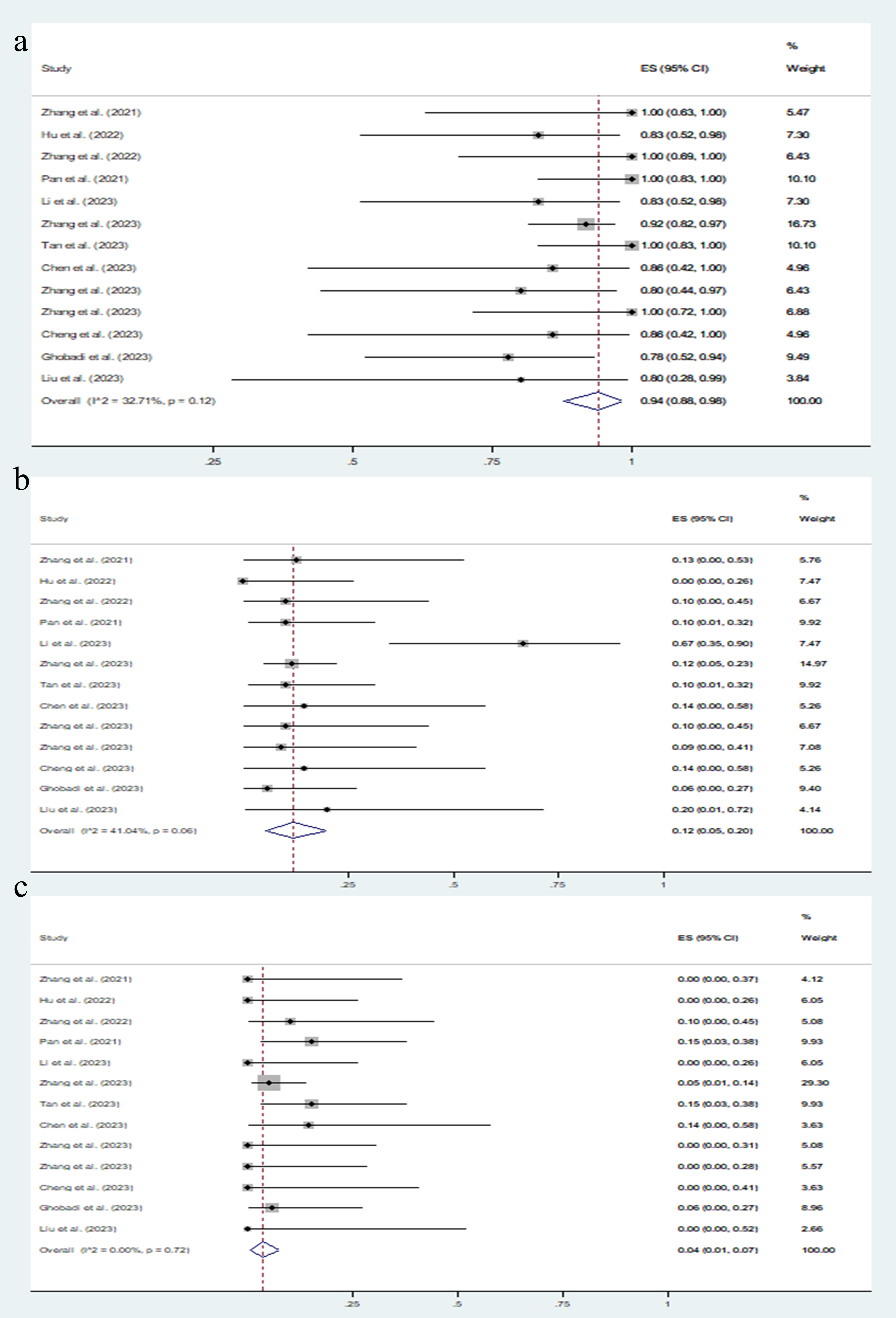

Supplement: Supplementary file 2 [file Image2.tif]
